# Mortality and loss-to-follow-up during the pre-treatment period in an antiretroviral therapy programme under normal health service conditions in Uganda

**DOI:** 10.1186/1471-2458-9-290

**Published:** 2009-08-11

**Authors:** Barbara Amuron, Geoffrey Namara, Josephine Birungi, Christine Nabiryo, Jonathan Levin, Heiner Grosskurth, Alex Coutinho, Shabbar Jaffar

**Affiliations:** 1 MRC/UVRI Uganda Research Unit on AIDS, c/o Medical Research Council (MRC UK), Uganda; 2 Uganda Virus Research Institute (UVRI) PO Box 49, Entebbe, Uganda; 3 The AIDS Support Organisation, Old Mulago Complex, PO Box 10443, Kampala, Uganda; 4 Department of Epidemiology and Population Health, London School of Hygiene & Tropical Medicine, Keppel Street, London, WC1E 7HT, UK; 5 Infectious Disease Institute, Mulago Hospital Complex, PO Box 22418, Kampala, Uganda

## Abstract

**Background:**

In many HIV programmes in Africa, patients are assessed clinically and prepared for antiretroviral treatment over a period of 4–12 weeks. Mortality rates following initiation of ART are very high largely because patients present late with advanced disease. The rates of mortality and retention during the pre-treatment period are not well understood. We conducted an observational study to determine these rates.

**Methods:**

HIV-infected subjects presenting at The AIDS Support Clinic in Jinja, SE Uganda, were assessed for antiretroviral therapy (ART). Eligible subjects were given information and counselling in 3 visits done over 4–6 weeks in preparation for treatment. Those who did not complete screening were followed-up at home. Survival analysis was done using poisson regression.

**Results:**

4321 HIV-infected subjects were screened of whom 2483 were eligible for ART on clinical or immunological grounds. Of these, 637 (26%) did not complete screening and did not start ART. Male sex and low CD4 count were associated independently with not completing screening. At follow-up at a median 351 days, 181 (28%) had died, 189 (30%) reported that they were on ART with a different provider, 158 (25%) were alive but said they were not on ART and 109 (17%) were lost to follow-up. Death rates (95% CI) per 100 person-years were 34 (22, 55) (n.18) within one month and 37 (29, 48) (n.33) within 3 months. 70/158 (44%) subjects seen at follow-up said they had not started ART because they could not afford transport.

**Conclusion:**

About a quarter of subjects eligible for ART did not complete screening and pre-treatment mortality was very high even though patients in this setting were well informed. For many families, the high cost of transport is a major barrier preventing access to ART.

## Background

Antiretroviral therapy has been scaled-up rapidly in Africa and elsewhere [1]. Early reports suggest that mortality of HIV-infected on ART is typically between about 6 to 12 deaths per 100 person-years [2-7], which is substantially better than in the pre-ART era [8] but higher than in developed countries [6]. One reason for the higher mortality in Africa is that patients in Africa present late for treatment, typically at 100–150 × 10^6^/l CD4 cell count lower than in developed countries and with advanced disease [9]. Mortality in Africa is especially high during the first year after initiation of ART[10]. Retention of subjects in ART programmes in Africa is also relatively low [11], suggesting that the burden of mortality is underestimated in some settings.

In many programmes in Africa, patients are assessed clinically and prepared for treatment over a period of 4–12 weeks [9,12]. They typically attend clinic on three occasions when information and counselling is provided on adherence and other aspects of ART. Pre-treatment mortality of around 30 deaths per 100 person-years has been reported from 2 studies in South Africa [13-15], but very little is known about rates of mortality elsewhere or patient retention around this time, especially in routine service delivery settings. We report the findings of a study which screened over 4000 subjects for ART and established survival rates and other outcomes among the subjects who were eligible for ART but dropped out of the programme and did not start treatment.

## Methods

The study was conducted at The AIDS Support Organisation (TASO) clinic in Jinja, SE Uganda. TASO is a large non-governmental organisation with 11 centres in the country offering counselling, social and clinical services to HIV-infected subjects.

The clinic in Jinja serves a predominantly rural/semi-urban population up to a radius of about 100 kilometres. Treatment and care are provided free and patients are managed according to national health service guidelines.

TASO began providing information about ART to its clients around the end 2003, mostly through group meetings and drama shows. Patients were screened for eligibility from August 2004 and the first patients began treatment about 4 weeks later. Recruitment into the programme ended in December 2006 when the funds available at that time had been used up. TASO was by far the largest provider of ART in the region. Patients who had been with the organisation the longest were given priority during the first year of the programme and thereafter ART was made available to all on a first come first served basis.

Assessment for ART was done over 3 visits to clinic which were usually spread over 4–8 weeks. At the first visit, patients were examined clinically and blood was taken and sent for CD4 count testing. Information and counselling were provided on ART focussing on adherence, side-effects, drug sharing, safer sexual behaviour, and was done both one-to-one and in groups. The second appointment was usually 2 weeks later. Those at WHO stage III (chronic fever of unknown origin, chronic diarrhoea of unknown origin, oral hairy leukoplakia and pyomyositis), stage IV, or with CD4 count less 200 × 10^6^/l were considered eligible for ART. Counselling was repeated and patients were asked to return for a 3^rd ^visit approximately 2 weeks later. They were also asked to identify a medicine companion who could provide support and reminders about drug taking. Between the 2^nd ^and 3^rd ^visit, case-conferences were held by TASO clinical and counselling staff. Patients' readiness for ART as well as their clinical eligibility was re-assessed and a decision was taken whether or not to provide ART. It was then at the 3^rd ^visit that ART was offered (following further information and patient counselling). The first line regimen comprised either zidovudine or stavudine, lamivudine, and either nevirapine or efavirenz. Those who were not eligible for ART (at the second visit) were provided counselling and asked to return to clinic every 3–6 months for monitoring.

Patients who completed the screening and started on ART after February 2005 were invited to join a cluster randomised trial comparing different ART delivery strategies, which was co-ordinated from the study site [12,16,17]. The trial was done under normal programme conditions with TASO staff responsible for patient screening before and patient management during the trial whilst a team of independent research staff documented trial outcomes. Trial and non-trial subjects were managed identically and for example no incentives were given either to staff or patients.

The data presented here refer to the screening period before ART initiation and were collected to inform TASO services. Follow-up of subjects who were eligible for ART but dropped out of the programme and did not initiate on ART at TASO Jinja was done between June 2007 and December 2007. We ascertained survival status and if alive, whether or not the subject was on ART from a different provider. Visits were done by trained field officers. Deaths were verified by close family members. Subjects not found at home were visited again at least once.

Distributions of sex, age and CD4 count categories were compared between groups using a chi-squared test. Comparions of CD4 count continuous data were compared using the Kruskall-Wallis test. Factors associated with completing three screening visits and starting ART were investigated by fitting logistic regression models. The analysis of survival time (time to death or date last seen alive) was investigated using survival analysis methods, with the survival time being described using a Kaplan Meier plot and summarized using mortality rates and incidence rates for a composite endpoint of mortality or loss-to-follow-up. The effect of CD4 count on mortality was investigated by fitting Poisson regression models. All analyses were done using Stata release 10.0.

## Results

### Baseline characteristics

4321 subjects (74% women, 26% men) had an initial screen for eligibility for ART between September 04 and December 06. The median (IQR) CD4 cell count × 10^6^/l was 138 (78 to 286) between September 04 and December 04, 157 (69 to 305) between January 05 and December 05 and 222 (111 to 388) between January 06 and December 06. Overall, 1811 (42%) were at WHO clinical stage III or IV and 2267 (52%) had CD4 count < 200 × 10^6^/l; 2483 (57%) were eligible for ART either on clinical grounds or because they had CD4 count < 200 × 10^6^/l.

Only 2182/2483 (88%, 95% CI 87, 89) of subjects eligible for ART returned for a second visit and were informed of their eligibility for ART. The median time between the first and second visits was 14 days (IQR 14 to 21). 1846/2483 (74%, 95% CI 73 to 76%) of the eligible subjects returned for a third visit and started ART. The median interval between the initial screening and 3^rd ^visit was 33 (range 15 to 406) days. The median (IQR) baseline CD4 count (cells per 10^6^/l) of those who did not complete screening was 87 (25 to 149) compared with 116 (46 to 170) for those who completed screening (p < 0.001, Wilcoxon test) (Table 1). Male sex and low baseline CD4 count were associated independently with not completing screening; there was no association between age or WHO stage and screening completion (Table 1). The proportion eligible for treatment who did not complete screening increased over time: it was 50/304 (16%) between September 04 and December 04, 157/737 (21%) between January 05 and December 05 and 430/1442 (30%) between January 06 and December 06.

**Table 1:** Baseline characteristics of the subjects who completed screening over 3 visits and started on ART and those who did not

	Completed 3 screening visits and started ART		Odds ratio (95% CI) for not completing screening	
	
	No (n. 637)	Yes (n. 1846)	Univariate	Multivariate *
Men	211 (29)	520 (71)	1.26 (1.04, 1.54)	1.28 (1.05, 1.56)
women	426 (24)	1,326 (76)	1.0	1.0
			(p = 0.02)	(p = 0.02)
Age, years				
<33	213 (28)	538 (72)	1.23 (0.98, 1.53)	1.20 (0.95, 1.51)
33–39	183 (25)	561 (75)	1.01 (0.81, 1.27)	1.22 (0.98, 1.53)
≥ 40	241 (24)	747 (76)	1.0	1.0
			(p = 0.1)	(p = 0.2)
CD4 count × 10^6^/l				
<50	232 (32)	485 (68)	1.72 (1.40, 2.12)	1.31 (1.0, 1.71)
50–99	114 (27)	314 (73)	1.31(1.01, 1.69)	1.68 (1.37, 2.06)
≥100	291 (22)	1047 (78)	1.0	1.0
			(p < 0.0001)	(p < 0.001)
WHO stage	n = 628	n = 1842		
I, II	265 (24)	830 (76)	0.89 (0.74, 1.07)	-
III, IV	363 (26)	1,012 (74)	1.0	

Note: WHO stage was not significant in multivariate analysis (p = 0.5) and not included in the final model.

* adjusted for sex and age and WHO category

### Survival status of 637 subjects eligible for ART who did not complete screening

**Figure 1:**
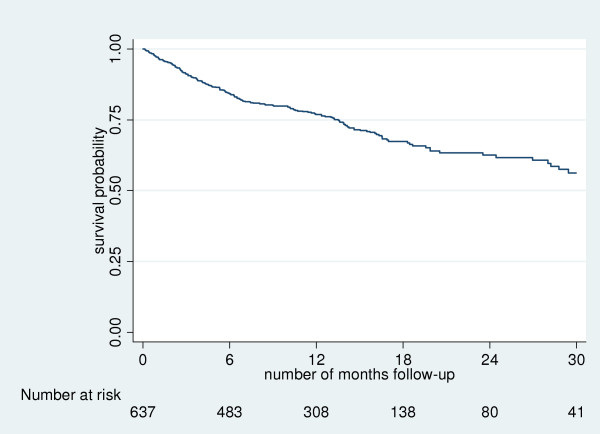
**Kaplan-Meier survival curve of 637 subjects who were eligible for antiretroviral therapy but did not complete screening and did not start on antiretrovirals at the TASO Jinja clinic**.

Subjects were followed-up at home to ascertain their survival status a median 351 (IQR 183 to 518) days from the first screening visit. One hundred and eighty-one (28%) had died, 189 (30%) reported they were now on ART but with a different provider, 158 (25%) were alive and but not on ART because of their choosing and 109 (17%) were lost to follow-up (Table 2) (Figure 1). Age and sex were not associated significantly with follow-up status but there were striking differences in baseline CD4 count: those who died or were lost to follow-up had substantially lower median CD4 counts than those who were alive and either not on ART or receiving this from another provider (Table 2).

**Table 2:** Factors associated with outcomes among 637 subjects who did not complete screening and did not start antiretroviral therapy despite being eligible for treatment on clinical grounds

	Died	On ART with another provider	Alive and not on ART	Lost to follow-up	p-value
	(n. 181)	(n. 189)	(n. 158)	(n. 109)	
Male, number (%)	68 (38)	65 (34)	47 (30)	31 (28)	0.3 *
					
Age ≤ 35 years, number (%)	81 (45)	91 (48)	72 (46)	63 (58)	0.15*
					
CD4 count × 10^6^/l					
Median (IQR)	38 (8, 102)	105 (37, 162)	129 (77, 172)	67 (10, 127)	0.0001^†^
					
<50	104 (57)	53 (28)	25 (16)	50 (46)	< 0.0001 *
50–99	30 (17)	37 (20)	29 (19)	18 (17)	
≥100	47 (26)	99 (52)	104 (66)	41 (38)	

* chi-squared test

† kruskall-wallis test

The mortality rate was 27 per 100 person-years (95% CI 23–31), while the rate for the composite endpoint of mortality or loss-to-follow-up was 43 per 100 person-years (95% CI 38–48). The rate ratios (95% CI) relative to subjects with CD4 count × 10^6^/l ≥100 were 2.82 (2.0, 4.0) among subjects with CD4 count <50 × 10^6^/l and 1.59 (1.0, 2.52) among subjects with CD4 count 50–99 × 10^6^/l. When stratified by calendar time, the mortality rates (95% CI) per 100 person-years were 34 (22, 55) (n.18) within one month, 37 (29, 48) (n.33) within 3 months, 35 (28, 42) (n. 97) within 6 months and 28 (24, 33) (n. 133) within 12 months.

Table 3 shows the reasons why the 158 subjects who were eligible for ART, did not start treatment and were traced at follow-up. Forty-four percent said that they did not start treatment because they could not afford transport costs. Just 4% reported difficulty in disclosure and declined to start ART for this reason.

**Table 3:** Reasons why antiretroviral therapy was not initiated in 158 subjects alive at follow-up and who were eligible for ART when screening was done

Subject could not afford transport costs (he/she had been screened in outreach centres near the home).	70 (44%)
Subject referred to another centre near home (e.g. because he/she requested or ART initiation was slow at TASO Jinja) but failed to turn up for treatment	6 (4%)
Subject said he/she was not ready to start life-long treatment	11 (7%)
Subject feared toxicity and side-effects of ART	2 (1%)
Subject failed to identify a medicine companion and did not return to the clinic	2 (1%)
Subject had difficulty in disclosing his/her HIV status and so did not complete the screening process.	7 (4%)
Subject was on TB treatment which he/she wanted to complete before starting ART	4 (3%)
Subject was not started on ART for a number of reasons (e.g. counsellor did not consider patient to be psychologically ready)	15 (9%)
Subject did not give a reason but continued visiting the centre for clinical follow-up	7 (4%)
Could not be traced at home (but was reported to be alive by family members).	34 (22%)

Note:status was assessed during home visits done a median of 351 days after initial screening. Subjects were asked for a single primary reason why they did not initiate ART.

## Discussion

Our study was conducted in a predominantly rural population which had been sensitised and was eagerly awaiting the introduction of ART. We found that a quarter of subjects did not complete screening with the service provider and did not initiate on ART. Of these, about half came once and never returned while half came for a second visit, were informed of their eligibility but despite learning this did not return again. The mortality rate during the screening procedures was very high, about 35 deaths per 100 person-years in the first month and similarly high shortly thereafter. This is consistent with findings from Cape Town, South Africa, where pre-treatment mortality was 30 deaths per 100-person-years [14,15]. In our study, about 17% of the subjects who had not completed screening could not be traced and they also had very low CD4 count at their first screening visit. This suggests that our pre-treatment mortality rate is probably an underestimate as many of these subjects may have died before they could access treatment elsewhere.

One major contributory factor to the high pre-treatment mortality is patients presented at an advanced HIV stage when serious co-morbidity is present, with low CD4 count, typically 100–150 cells per microlitre below when treatment is recommended. Not only is pre-treatment mortality high among such subjects, mortality shortly after ART initiation is also very high [10] and a major public health burden. Median CD4 counts of subjects at first presentation increased over the 29 months of screening during the study, suggesting that patients may have started to present at an earlier stage; however, the proportion of subjects eligible for ART who did not complete screening also increased, rising from 21% in 2004 to 30% in 2006. The reasons for this increase over time in the number who did not complete screening are unclear but it is possible that earlier patients were more motivated or that more recently there was a greater choice of service providers available and TASO patients left to join other programmes. What is clear is that mortality during screening is very high, especially among those presenting with low CD4 count.

About a third of subjects who did not complete screening were alive when they were followed-up at home about a year later. The median CD4 count at screening, which was done about one year earlier, was the highest among this group although still low at a median 129 × 10^6^/l CD4 cell counts and well below the target CD4 count for initiating ART. A staggering 44% of these said that they did not start ART because they were unable to afford transport fare. Our other research in this population suggests that family cash incomes are low, typically less than 10 Euros a month, and transport costs high, typically around 0.5 to 1 Euro each visit [12]. Thus costs are a major barrier for patients accessing long-term chronic care from rural settings such as ours. These must be taken into account in efforts to expand access to ART and sustain adherence. For this reason, one of TASO's strategies includes home based delivery of ART but this involves substantial costs for the provider and may not be sustainable or feasible in other parts of Africa. A proportion of those who died are likely to have had difficulty in accessing care because of costs and so we have probably underestimated the burden of high transport costs. Sustaining effective delivery of treatment to such populations and ensuring regular contact with health service staff to monitor and re-enforce adherence will be a major challenge. A strategy may be to establish outreach services for patient monitoring and drug refills that are delivered by skilled paramedical workers at larger rural centres sufficiently close to the communities where patients live. However, whether this could be effective needs evaluation.

In our setting, and indeed in other parts of Uganda, disclosure does not appear to be a major barrier to accessing treatment [12]. TASO provides assistance to patients to disclose where necessary and consequently just 4% of those not on ART said that this was because they had difficulty disclosing. We know in this and other settings in Uganda that fewer men than women are on antiretroviral treatment [12,18]. In this study, among those who had come forward to the health service, men were about 30% less likely to complete screening as women. The reasons for this are unclear and warrants investigation in this setting.

## Conclusion

This study shows that about a quarter of subjects eligible for ART did not complete screening and did not initiate on ART. The mortality rate was very high during the pre-treatment period. Almost half of those still alive at the follow-up visit said they had not completed screening because they could not afford the transport costs to come to the clinic. For many families, the high cost of transport is a major barrier preventing access to ART.

## Authors' contributions

BA co-ordinated field activities and wrote the first draft of the paper. GN and JL did the statistical analysis. JB was responsible for recruitment of subjects and patient management. CN and AC had the original idea and contributed to the design of the study. HG gave overall support. SJ planned the study and wrote subsequent drafts of the paper. All authors reviewed drafts critically and contributed to ideas and writing.

## Pre-publication history

The pre-publication history for this paper can be accessed here:


